# Serum Zonulin and Chitinase (CHI3L1) as Biomarkers of Intestinal Permeability and Disease Activity in Pediatric Celiac Disease

**DOI:** 10.3390/children13060730

**Published:** 2026-05-24

**Authors:** Ayşegül Cebe Tok, Oya Sayın

**Affiliations:** 1 Department of Pediatric Gastroenterology, Etlik City Hospital, University of Health Sciences, Ankara 06010, Türkiye; 2 Department of Medical Laboratory Techniques, Vocational School of Health Services, Dokuz Eylul University, Izmir 35340, Türkiye; oya.sayin@deu.edu.tr

**Keywords:** intestinal barrier function, mucosal injury, serological biomarkers, Marsh classification, gluten-free diet adherence

## Abstract

**Highlights:**

**What are the main findings?**

- Serum zonulin and CHI3L1 levels were lower in patients adherent to a gluten-free diet and higher in newly diagnosed and non-adherent patients.
- Serum zonulin and CHI3L1 levels showed positive correlations with tissue transglutaminase IgA (tTG-IgA) and histopathological severity, reflecting disease activity in pediatric celiac disease.

**What are the implications of the main findings?**

- Zonulin and CHI3L1 may serve as complementary biomarkers for monitoring dietary adherence and disease activity in pediatric celiac disease.
- These biomarkers may support conventional follow-up by reflecting intestinal inflammation and mucosal involvement.

**Abstract:**

**Objectives:** To evaluate serum zonulin and CHI3L1 as indicators of intestinal permeability and disease activity in pediatric celiac disease and to explore their associations with histopathological findings and nutritional status. **Methods:** This prospective cross-sectional study included 131 pediatric patients with CD (aged 2–18 years) and 42 healthy controls. Patients were classified as newly diagnosed, gluten-free diet (GFD)-adherent, or GFD-nonadherent. Body mass index was calculated, and serum levels of micronutrients, zonulin, and CHI3L1 were measured using a sandwich enzyme-linked immunosorbent assay. Associations with histopathological findings, serological markers, and nutritional parameters were analyzed. **Results:** Age and sex distributions were similar across groups (mean age: 10.9 ± 4.27 years). Serum zonulin and CHI3L1 levels were moderately positively correlated (r = 0.525, *p* < 0.001). Both biomarkers showed significant positive correlations with Marsh scores and tissue transglutaminase IgA levels. Zonulin was inversely correlated with hemoglobin, serum iron, and ferritin, whereas CHI3L1 showed negative correlations with hemoglobin and folate. Parathyroid hormone levels were positively correlated with both biomarkers. Receiver operating characteristic analysis demonstrated acceptable discriminatory performance for distinguishing CD from controls (AUC: 0.713 for zonulin and 0.709 for CHI3L1). **Conclusions:** Serum zonulin and CHI3L1 levels are associated with disease activity and mucosal injury in pediatric CD but do not directly reflect micronutrient status. These biomarkers may complement conventional monitoring parameters by providing additional information on intestinal permeability and inflammatory activity during follow-up.

## 1. Introduction

Celiac disease (CD) is a T-cell-mediated autoimmune disorder that develops in genetically predisposed individuals following gluten exposure and has a steadily increasing global prevalence [1,2]. Activation of gluten-specific T cells induces an inflammatory cascade in the intestinal mucosa, characterized by increased production of proinflammatory cytokines such as interleukin-15 (IL-15), interleukin-17A (IL-17A), and interferon-γ (IFN-γ) [3]. In parallel, gliadin activates a MyD88-dependent signaling pathway via the CXCR3 receptor on intestinal epithelial cells, leading to zonulin release and disruption of tight junction integrity [4]. This increased intestinal permeability facilitates the translocation of immunogenic gluten peptides across the epithelial barrier, triggering both innate and adaptive immune responses [5]. Zonulin expression is upregulated during active CD and may precede overt clinical or histopathological manifestations, suggesting its role in early disease pathogenesis [6,7].

Another pathway implicated in CD-related inflammation involves CHI3L1-3-like-1 (CHI3L1), a glycoprotein produced by epithelial and immune cells in response to cytokines such as IL-15 and IFN-γ [8]. Elevated CHI3L1 levels promote chemotaxis and accumulation of antigen-presenting cells in the lamina propria and are associated with tissue remodeling, angiogenesis, and chronic inflammatory activity [9,10,11]. Accordingly, CHI3L1 is considered a biomarker of inflammatory and endothelial dysfunction [12].

Although a gluten-free diet (GFD) remains the cornerstone of CD management, mucosal healing is heterogeneous and may be delayed despite adherence to the diet [13]. Persistent villous atrophy and residual inflammation have been reported in a subset of patients, particularly those with advanced Marsh classification, even when serological markers improve [14,15,16]. Moreover, long-term GFD may not fully prevent nutritional deficiencies, and abnormalities in BMI, hematologic indices, and micronutrient levels have been documented despite compliance [17,18,19].

Serum zonulin has been proposed as a non-invasive marker of intestinal permeability in celiac disease, with some studies reporting higher levels in active disease and potential associations with mucosal damage. However, its clinical utility remains controversial due to inconsistent findings and concerns regarding the specificity of available commercial assays. Similarly, chitinase-3-like protein 1 (CHI3L1) has been suggested as an inflammatory marker, but its role in pediatric celiac disease is not yet well defined. In this pediatric cohort, we aimed to evaluate serum zonulin and CHI3L1 levels in relation to gluten-free diet adherence and clinical/laboratory parameters, to better clarify their potential role in disease follow-up.

Given the limitations of conventional follow-up parameters, there is a growing need for complementary biomarkers that better reflect ongoing intestinal barrier dysfunction and inflammation. Therefore, this study aimed to evaluate the relationship between persistent micronutrient abnormalities and inflammatory activity in pediatric CD and to assess whether zonulin and CHI3L1 can serve as adjunctive biomarkers independent of tissue transglutaminase IgA.

## 2. Materials and Methods

### 2.1. Ethics Statement

This study was approved by the Institutional Ethics Committee of Etlik City Hospital (Approval No: AEŞH-BADEK-2024-937; Date: 12 March 2025). Written informed consent was obtained from the parents or legal guardians of all participants in accordance with the Declaration of Helsinki.

### 2.2. Study Design and Population

This prospective, cross-sectional study was conducted between 2024 and 2025 at the Pediatric Gastroenterology Outpatient Clinic of Etlik City Hospital. A total of 173 participants were enrolled after obtaining informed consent from their legal guardians. The study population was divided into two primary groups: the Celiac Disease (CD) group (*n* = 131) and the Healthy Control (HC) group (*n* = 42). The CD group was further categorized into three subgroups based on clinical status and dietary adherence as follows:

- Newly Diagnosed (*n* = 35): Patients recently diagnosed and evaluated prior to the initiation of a gluten-free diet (GFD).
- GFD-Compliant (*n* = 58): Patients who demonstrated strict adherence to the GFD for at least one year.
- GFD-non-compliant (*n* = 38): Patients who did not adhere to dietary restrictions.

### 2.3. Diagnosis and Adherence Assessment

The diagnosis of CD was established according to the 2012 ESPGHAN criteria, confirmed by positive anti-tissue transglutaminase IgA (anti-tTG IgA) titers (>20 IU/L) and histopathological validation via duodenal biopsy. Dietary adherence was comprehensively evaluated by a specialized dietitian based on (1) detailed nutritional anamnesis, (2) label-reading behavior and awareness, (3) assessment of cross-contamination risks, and (4) the longitudinal course of anti-tTG IgA levels over the preceding six months.

### 2.4. Control Group and Inclusion/Exclusion Criteria

The healthy control group consisted of 42 children who presented with minor complaints such as functional constipation, reflux, or non-specific abdominal pain, but were otherwise healthy. These individuals had no chronic diseases, no signs of acute infection, and no history of antibiotic use in the previous two months.

The exclusion criteria for all participants were as follows:

- C-reactive protein (CRP) levels >10 mg/L.
- Concurrent infections (e.g., urinary tract infection, diarrhea, upper respiratory tract infection).
- Antibiotic use within the last two months.
- Lack of informed consent.

### 2.5. Laboratory Measurements and Sample

Processing Anthropometric measurements were recorded, and Body Mass Index (BMI) was calculated for all participants. Venous blood samples were collected to evaluate the complete blood count, thyroid function, total IgA, anti-tTG IgA and IgG, iron, ferritin, vitamin B12, folate, calcium, magnesium, phosphorus, vitamin D, and parathyroid hormone (PTH) levels.

For the analysis of serum zonulin and CHI3L1-1 (CHI3L1), blood samples were centrifuged at 3000 rpm for 10 min within 60 min of collection. The separated serum aliquots were immediately stored at −80 °C until further analysis.

### 2.6. Biochemical Analysis of Zonulin and CHI3L1-1

Serum concentrations of zonulin and CHI3L1 were quantified using commercial human enzyme-linked immunosorbent assay (ELISA) kits: Human Zonulin ELISA Kit (Cat. No. ABT3174Hu, A.B.T. Laboratory Industry, Ankara, Türkiye) and Human CHI3L1 ELISA Kit (Cat. No. ABT2703Hu, A.B.T. Laboratory Industry, Ankara, Türkiye). Both assays utilized a sandwich ELISA methodology with monoclonal antibodies and horseradish peroxidase (HRP) conjugation.

- Zonulin Assay (Cat. No. ABT3174Hu): Detection range 0.79–50 ng/mL; sensitivity 0.47 ng/mL.
- CHI3L1 Assay (No. ABT2703Hu): Detection range 78.13–5000 pg/mL; sensitivity 46.88 pg/mL.

Both assays maintained intra- and inter-assay coefficients of variation (CV) of <10%. Technical Note: As commercial zonulin ELISA kits primarily recognize zonulin-related peptides rather than the authentic pre-haptoglobin-2 molecule, the results of this study were interpreted as relative indicators of intestinal permeability rather than absolute protein quantification.

### 2.7. Statistical Analyses

Statistical analyses were performed using SPSS Statistics version 26.0 (IBM Corp., Armonk, NY, USA). Descriptive statistics are presented as mean ± standard deviation or median (minimum–maximum) for continuous variables and as frequencies (*n*) and percentages (%) for categorical variables. The normality of the data was evaluated using the Shapiro–Wilk test, skewness–kurtosis values (thresholds: skewness < |2| and kurtosis < |7|), and visual inspection of histograms.

- Parametric Data: Analyzed using one-way analysis of variance (ANOVA) followed by Tukey’s post hoc test.
- Non-parametric Data: Variables such as zonulin, CHI3L1-1, ferritin, and specific leukocyte counts were analyzed using the Kruskal–Wallis test, with Dunn–Bonferroni post hoc analysis for multiple comparisons.
- Categorical Data: Compared using the chi-square test.
- Correlation: Pearson or Spearman correlation coefficients were used, defined as low (0.00–0.30), moderate (0.30–0.70), or high (0.70–1.00). Statistical significance was set at *p* < 0.05.

## 3. Results

### 3.1. Demographic Characteristics and Nutritional Status

The study population consisted of 173 participants, with a higher proportion of females (64.2%, *n* = 111) than males (35.8%, *n* = 62). The mean age was 10.9 ± 4.27 years (range, 2–18 years; median, 11 years). No statistically significant differences were observed between the study groups in terms of age and sex distribution (*p* > 0.05).

Regarding nutritional status, BMI-SDS values were significantly lower in all Celiac Disease (CD) subgroups than in the healthy control (HC) group. While 74.4% of healthy controls had a BMI within the normal range, this rate dropped to 40.5% in patients with non-compliant GFD. In the newly diagnosed group, nine patients (seven males and two females) had BMI-SDS values below −2. Post hoc analysis (Tukey HSD) revealed that the BMI values in the HC group were significantly higher than those in the newly diagnosed (*p* = 0.021), GFD-compliant (*p* = 0.001), and GFD-non-compliant (*p* = 0.020) groups. However, no significant differences were observed among the three CD subgroups (*p* > 0.05).

### 3.2. Hematologic and Biochemical Parameters

Significant differences were identified across the groups in several hematologic and micronutrient parameters (Table 1). Hemoglobin, hematocrit, MCV, and MCHC levels were significantly lower in newly diagnosed and GFD-non-compliant patients than in the control group. Similarly, ferritin, Ca, and Mg levels were significantly reduced in the CD subgroups (Table 1).

The independent-samples median test demonstrated statistically significant differences in the evaluated biomarkers between the study groups (*p* < 0.05). The results of the pairwise comparisons and the corresponding letters used to denote group differences are presented in Table 1. While zonulin and CHI3L1 levels, similar to tTG-IgA, were low in the control and GFD-compliant groups, they were significantly elevated in newly diagnosed and non-compliant patients. Folate and Vitamin B12 levels did not differ significantly between the groups. Regarding calcium metabolism, serum calcium and magnesium levels were lower in newly diagnosed and non-compliant patients, whereas a heterogeneous distribution was observed in the GFD-compliant group. Parathyroid hormone (PTH) levels were significantly higher in the GFD-non-compliant patients (Table 1).

### 3.3. Distribution of Serum Zonulin and Chitinase (CHI3L1) Levels Across the Study Groups

**Figure 1:**
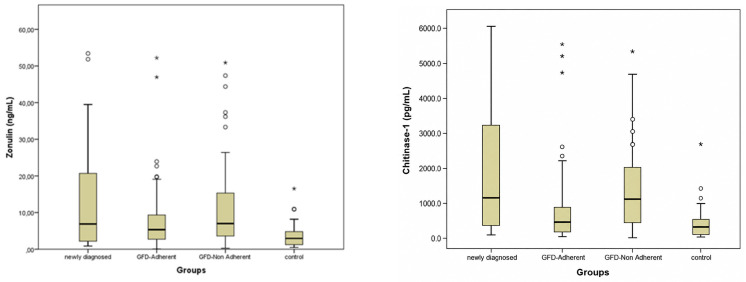
Zonulin and Chitinase-1 (CHI3L1) Levels of the Groups. Circles (○) indicate outliers and asterisks (*) *indicate extreme outliers*.

Figure 1 illustrates the distribution of serum zonulin and chitinase (CHI3L1) levels across the study groups, with higher levels observed in the newly diagnosed and non-adherent groups compared to the adherent and control groups.

### 3.4. Zonulin and CHI3L1-1 Levels and Histopathology

When stratified according to Marsh classification, serum zonulin and CHI3L1-1 levels demonstrated comparable distributions across histological subgroups. Median zonulin levels were 5.54 ng/mL (IQR: 2.24–19.76) in Marsh 2, 7.29 ng/mL (IQR: 4.34–14.46) in Marsh 3a, 5.60 ng/mL (IQR: 2.70–10.31) in Marsh 3b, and 6.25 ng/mL (IQR: 3.04–10.77) in Marsh 3c. No statistically significant differences in zonulin levels were observed among the Marsh subgroups (*p* = 0.937) (Supplementary Table S1).

Similarly, CHI3L1-1 levels did not differ significantly across histological stages, with median values of 1080 ng/mL (IQR: 217–1851) in Marsh 2, 642 ng/mL (IQR: 213–1851) in Marsh 3a, 802 ng/mL (IQR: 321–1444) in Marsh 3b, and 733 ng/mL (IQR: 424–1943) in Marsh 3c (*p* = 0.843) (Supplementary Table S1).

### 3.5. Correlation and Diagnostic

Performance Zonulin and CHI3L1 levels did not directly correlate with age or BMI. However, both biomarkers demonstrated significant positive correlations with the Marsh score (zonulin: r_s_ = 0.289, *p* < 0.001; CHI3L1: r_s_ = 0.243, *p* = 0.001) and tTG-IgA levels (zonulin: r_s_ = 0.245, *p* = 0.001; CHI3L1: r_s_ = 0.264, *p* < 0.001) (Table 2).

### 3.6. Hematologic and Iron Profiles:

Zonulin levels showed significant negative correlations with hemoglobin (r_s_ = −0.258), Serum Iron (r_s_ = −0.203), and ferritin (r_s_ = −0.202) (*p* < 0.01). Similarly, CHI3L1 was inversely correlated with hemoglobin (r_s_ = −0.160) and folate (r_s_ = −0.180) levels (Table 2).

Metabolic and Hormonal Indicators: PTH levels, reflecting bone mineral metabolism disturbances, were positively correlated with zonulin (r_s_ = 0.203, *p* = 0.010) and CHI3L1 (r_s_ = 0.190, *p* = 0.015 (Table 2).

**Figure 2:**
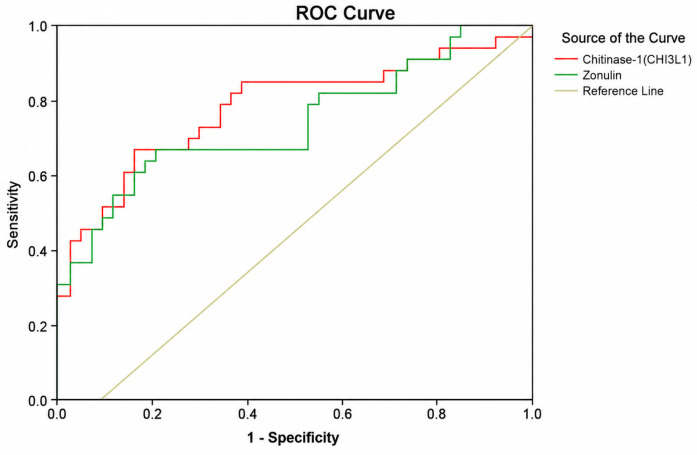
Receiver Operating Characteristic (ROC) Curves for Zonulin and Chitinase-1 (CHI3L1) in Discriminating Celiac Disease from Healthy Controls. The Red Line Represents Chitinase-1(CHI3L1) And The Green Line Represents Zonulin. The Diagonal Line Indicates The Reference Line (Auc = 0.50).

ROC analysis demonstrated that zonulin had an AUC of 0.713 (95% CI: 0.630–0.797, *p* < 0.001), with an optimal cut-off value of 1.53 determined by the Youden index, yielding 85.9% sensitivity and 65.8% specificity (Youden index = 0.517). Chinitase showed a similar AUC of 0.709 (95% CI: 0.623–0.796, *p* < 0.001), but with lower diagnostic performance. The optimal cut-off value was 49.5 based on the Youden index, yielding 91.1% sensitivity but only 28.9% specificity (Youden index = 0.200) (Figure 2).

## 4. Discussion

Current clinical observations in celiac disease (CD) indicate that some patients continue to exhibit micronutrient deficiencies and low-grade inflammation despite adherence to a gluten-free diet (GFD) [15]. This suggests that conventional follow-up parameters, including tissue transglutaminase IgA (tTG-IgA) and symptom assessment, may not fully reflect ongoing disease activity. Therefore, interest has increased in biomarkers related to intestinal barrier dysfunction and mucosal inflammation [19]. In this study, we evaluated zonulin and CHI3L1 levels across different clinical CD groups and investigated their associations with micronutrient deficiencies, inflammatory activity, and histopathological findings.

### 4.1. Intestinal Barrier Dysfunction and Zonulin

In celiac disease, gluten ingestion triggers enterocyte injury and activates both innate and adaptive immune pathways. Gliadin peptides stimulate MyD88-dependent inflammatory signaling, likely through Toll-like receptor activation, while simultaneously inducing zonulin release via CXCR3 engagement [3,20]. Increased zonulin expression disrupts tight junction integrity and increases intestinal permeability, facilitating the translocation of gluten-derived peptides and microbial products such as lipopolysaccharide (LPS) into the lamina propria [4,21,22]. This process contributes to persistent mucosal immune activation and amplification of the inflammatory response.

Zonulin is recognized as a key regulator of intestinal barrier function, and elevated levels have consistently been demonstrated in patients with celiac disease [5,22]. Beyond its mechanistic role in barrier dysfunction, recent studies suggest that zonulin may also serve as a biomarker reflecting disease development and progression [6,23]. Experimental studies further support the relationship between epithelial integrity and inflammatory activity, demonstrating that increased expression of tight junction proteins such as Zonula Occludens-1 (ZO-1) is associated with lower production of pro-inflammatory cytokines including TNF-α and IL-6.

Barrier dysfunction also alters the interaction between the gut microbiota and the host immune system. Under physiological conditions, microbiota-derived short-chain fatty acids (SCFAs), particularly butyrate, support peripheral regulatory T-cell differentiation through mechanisms involving histone deacetylase inhibition and FoxP3 induction, thereby maintaining mucosal tolerance [1,2,24,25]. However, increased intestinal permeability and dysbiosis disrupt this regulatory balance and promote pro-inflammatory immune responses.

In celiac disease, gluten ingestion triggers enterocyte injury and activates both innate and adaptive immune pathways. Gliadin peptides induce inflammatory signaling and zonulin release via CXCR3 engagement, leading to disruption of tight junction integrity and increased intestinal permeability [3,20,21,22]. This process facilitates the translocation of gluten-derived peptides and microbial products into the lamina propria, contributing to persistent mucosal immune activation.

Zonulin is recognized as a key regulator of intestinal barrier function, and elevated levels have consistently been demonstrated in patients with celiac disease [5,22]. Beyond its mechanistic role, zonulin may also reflect disease activity and progression [6,23]. Experimental evidence further supports the relationship between epithelial integrity and inflammation, as preservation of tight junction function has been associated with lower pro-inflammatory cytokine activity.

Intestinal barrier dysfunction may also alter host–microbiota interactions. Microbiota-derived short-chain fatty acids, particularly butyrate, contribute to mucosal immune tolerance through regulatory T-cell pathways, whereas increased permeability and dysbiosis may shift this balance toward pro-inflammatory responses [1,2,24,25]. In line with these mechanisms, serum zonulin levels were significantly elevated in our cohort, particularly in newly diagnosed and non-compliant patients, supporting the presence of ongoing barrier dysfunction.

### 4.2. CHI3L1 and Innate Immune Activation

Within this inflammatory milieu, CHI3L1 appears to function not only as a biomarker but also as a mediator involved in innate immune activation and tissue remodeling. Increased CHI3L1 expression in celiac intestinal tissue has been associated with IL-15Rα and Tissue Transglutaminase 2 (TGM2) expression, suggesting a role in amplification of inflammatory signaling pathways [7]. In addition, CHI3L1 has been implicated in chronic inflammation and tissue remodeling processes in several inflammatory disorders [9,10,11,26,27,28]. Collectively, these findings suggest that zonulin-mediated barrier dysfunction, microbiota alterations, and CHI3L1-associated innate immune activation interact within an integrated mucosal network contributing to the pathogenesis and persistence of celiac disease.

### 4.3. Clinical Findings and Micronutrient Deficiencies

Growth and developmental retardation are classic early manifestations of CD. Monzani et al.’s 30-year retrospective analysis reported a decrease in the rate of BMI < −2 SDS from 24.4% (1990–2011) to 12.7% in the following decade, attributed to earlier diagnosis and increased clinical awareness [29]. In our study, 25% of newly diagnosed patients had BMI < −2 SDS, consistent with Monzani’s earlier data. However, this rate may not represent the broader population due to sample size limitations and rising obesity prevalence. The lower BMI-SDS distribution in all CD subgroups compared to healthy controls, and the significantly lower average BMI-SDS in non-compliant patients compared to those adhering to the GFD, support the adverse effect of continued gluten exposure on growth.

Growth retardation remains a recognized manifestation of pediatric celiac disease. In our study, 25% of newly diagnosed patients had BMI < −2 SDS, consistent with previous pediatric reports [29]. Lower BMI-SDS values across CD subgroups, particularly among non-compliant patients, support the adverse impact of continued gluten exposure on growth.

Iron deficiency anemia (IDA), resulting from mucosal damage, is one of the most common clinical findings in CD. Our results confirm significantly lower hemoglobin, hematocrit, MCV, and ferritin levels in CD groups versus healthy controls (Table 1). Moreover, ferritin, iron, hemoglobin, and MCHC negatively correlated with zonulin, linking increased intestinal permeability to anemia, a hallmark of CD (Table 2). IDA prevalence in newly diagnosed CD ranges from 25% to 82% [30,31,32]. Sanseviero et al. reported iron deficiency and IDA as common at diagnosis in a pediatric cohort [32]. Persistent iron deficiency despite GFD may relate to permanent ultrastructural changes in enterocytes [33], although ferritin levels can normalize over time in adherent patients without supplementation [34]. In our cohort, hemoglobin levels approached those of healthy controls after at least one year on the diet. The moderate negative correlation between zonulin and anemia markers suggests ongoing increased intestinal permeability in patients with persistent IDA.

Micronutrient deficiencies, including iron, calcium, and Vitamin D, are frequent in CD. Although GFD improves mucosal atrophy and nutrient absorption, some mineral and vitamin levels may not fully normalize [35,36,37]. Kreutz et al. emphasized that many children with CD exhibit at least one nutrient deficiency during long-term follow-up, with Vitamin D and iron most common [38].

Micronutrient deficiencies, particularly involving iron and vitamin D, are common in celiac disease and may persist despite adherence to a gluten-free diet [35,36,37,38]. Persistent villous damage in the proximal small intestine may directly impair mineral absorption. Calcium deficiency in untreated pediatric CD varies widely (0–41%) [39,40]. In our study, serum calcium and magnesium levels were variable across groups, with no significant increase following dietary adherence. Although negative trends were observed between zonulin/CHI3L1 and calcium, phosphorus, or Vitamin D, these correlations did not reach statistical significance (Table 2).

Vitamin D plays a critical role in intestinal diseases involving malabsorption, being primarily absorbed in the duodenum and jejunum [41,42]. However, studies on Vitamin D levels in CD show inconsistent results. Some report no significant difference between new cases and controls [43,44,45], while others find lower levels at diagnosis, especially in children and adolescents [46]. Our study found no significant differences between groups; however, borderline low Vitamin D levels in healthy controls suggest a wider public health concern [47]. Elevated parathyroid hormone (PTH) levels in non-compliant patients, positively correlated with zonulin and CHI3L1, indicate that intestinal permeability and inflammation may indirectly affect calcium metabolism [48,49].

However, studies on vitamin D levels in celiac disease have reported inconsistent findings, with some showing no significant differences between newly diagnosed patients and controls, while others reported lower levels at diagnosis, particularly in children and adolescents [43,44,45,46]. Similarly, we found no significant differences between groups; however, borderline low vitamin D levels in healthy controls may reflect a broader public health concern [47]. In contrast, elevated parathyroid hormone (PTH) levels in non-compliant patients and their positive correlation with zonulin and CHI3L1 suggest that intestinal permeability and inflammation may indirectly influence calcium metabolism [48,49].

Malabsorption of folic acid and vitamin B12, absorbed in the jejunum and terminal ileum, respectively, may contribute to anemia in CD. Despite contradictory literature findings [49,50,51], our study found no significant differences in serum folic acid and vitamin B12 between CD subgroups and controls. A recent meta-analysis reported a lower risk of deficiencies in vitamin D, B12, E, calcium, and iron in patients adhering to GFD compared to untreated patients, though evidence quality was very low [51].

Although folic acid and vitamin B12 malabsorption may contribute to anemia in CD, we observed no significant differences between CD subgroups and healthy controls, in line with the inconsistent findings reported in the literature [49,50,51].

### 4.4. Marsh/Histopathology Interpretation

Zonulin is a key regulator of intestinal barrier integrity and is released following gliadin exposure, leading to increased intestinal permeability [3,5,20]. Previous studies demonstrated that zonulin levels may increase before the development of overt villous atrophy and celiac-specific autoantibodies, suggesting a role in early disease activity [6,21,22]. Consistent with these findings, serum zonulin levels were significantly elevated in our CD cohort, particularly in newly diagnosed and non-compliant patients, supporting the presence of ongoing barrier dysfunction.

Although zonulin and CHI3L1 levels showed significant positive correlations with both Marsh score and tTG-IgA, no significant differences were observed among Marsh subgroups (2, 3a, 3b, and 3c) (Supplementary Table S1). This finding may reflect the different temporal dynamics of circulating biomarkers and histopathological changes. While circulating biomarkers primarily indicate ongoing inflammatory and immune activity, Marsh classification reflects cumulative structural injury developing over time. Since zonulin-mediated barrier dysfunction occurs early in disease pathogenesis, biomarker elevations may already be present before advanced villous atrophy develops, potentially explaining the limited discrimination between Marsh subgroups despite significant overall correlations [6,21,22]. Furthermore, previous studies have shown that mucosal immune activation may persist despite partial histological recovery, suggesting that circulating biomarkers may better reflect active inflammation than structural damage alone [12].

The positive correlation observed with tTG-IgA is biologically relevant, as anti-TG2 immune activity has been shown to correlate with mucosal inflammation and tissue injury in celiac disease [13,14]. In addition, histopathological grading may be affected by the patchy distribution of mucosal lesions and sampling variability inherent to duodenal biopsies, which may further weaken correlations between circulating biomarkers and Marsh subgroups [12,14]. Consistent with this concept, pediatric studies evaluating zonulin have generally reported weak-to-moderate correlations with Marsh stages despite higher levels in Marsh 2–3 lesions [23,52].

This discrepancy may reflect differences between circulating biomarkers and histopathological injury. While biomarkers indicate ongoing immune and inflammatory activity, Marsh classification reflects cumulative structural damage. Since barrier dysfunction occurs early in celiac disease, biomarker elevations may precede advanced villous atrophy, potentially explaining the limited discrimination between Marsh subgroups despite significant overall correlations [6,12,21,22]. The positive correlation observed with tTG-IgA is biologically relevant, as anti-TG2 immune activity has been associated with mucosal inflammation and tissue injury in celiac disease [13,14]. In addition, the patchy distribution of mucosal lesions and sampling variability in duodenal biopsies may weaken correlations between circulating biomarkers and Marsh stages. Consistent with this concept, pediatric studies evaluating zonulin have generally reported weak-to-moderate correlations with Marsh stages despite higher levels in Marsh 2–3 lesions [12,14,23,52].

This study is the first to systematically evaluate zonulin, the primary regulator of intestinal permeability, and CHI3L1, an inflammatory marker, concurrently, along with their clinical impact in CD. While CHI3L1′s association with mucosal inflammation and macrophage activation is well-established in inflammatory bowel diseases (IBD) [8,48], no prior studies have directly measured serum CHI3L1 in CD or linked it to disease activity. Our findings show significant positive correlations of zonulin and CHI3L1 with tTG-IgA and Marsh score (*p* < 0.001), supporting the hypothesis that loss of barrier integrity and macrophage-mediated inflammation are interconnected in CD immunopathogenesis. The absence of significant differences between Marsh subgroups for zonulin and CHI3L1 suggests these markers peak early (by Marsh 2) and plateau as tissue damage progresses. CHI3L1 levels rise rapidly during acute inflammation but may stabilize or fluctuate in chronic or heterogeneous tissue damage [26,27,28]. These findings indicate zonulin and CHI3L1 are sensitive biochemical indicators for early diagnosis and mucosal integrity assessment rather than grading morphological severity. No significant correlations were found between systemic inflammation markers (WBC, lymphocytes) and zonulin or CHI3L1 (*p* > 0.05). However, a weak but significant positive correlation between peripheral monocyte count and zonulin (r = 0.154, *p* = 0.046) suggests increased intestinal permeability allows antigen translocation stimulating the monocyte-macrophage axis. The lack of strong associations with hematological parameters and Marsh stages confirms CD as an organ-specific, chronic, low-grade inflammatory disease rather than a systemic infection.

This study is the first to systematically evaluate zonulin and CHI3L1 concurrently, together with their clinical relevance in celiac disease. While CHI3L1 has been associated with mucosal inflammation and macrophage activation in inflammatory bowel diseases (IBD) [8,48], no previous studies have directly evaluated serum CHI3L1 in CD or its association with disease activity. In our study, zonulin and CHI3L1 showed significant positive correlations with tTG-IgA and Marsh score (*p* < 0.001), supporting the link between barrier dysfunction and inflammatory activity in CD. However, the absence of significant differences between Marsh subgroups suggests that these biomarkers may reflect early mucosal changes rather than the severity of histopathological damage. CHI3L1 levels may rise during acute inflammation but stabilize in chronic or heterogeneous tissue injury [26,27,28].

Zonulin and CHI3L1 may therefore serve as sensitive biochemical indicators of mucosal integrity and inflammatory activity rather than morphological severity. No significant correlations were observed between systemic inflammatory markers and zonulin or CHI3L1, although the weak positive correlation between peripheral monocyte count and zonulin (r = 0.154, *p* = 0.046) may reflect low-grade immune activation associated with increased intestinal permeability. Overall, the limited associations with hematological parameters and Marsh stages support CD as a chronic, organ-specific, low-grade inflammatory disease rather than a systemic inflammatory condition.

### 4.5. Diagnostic Performance of Zonulin and CHI3L1

ROC analysis demonstrated moderate diagnostic performance for zonulin and CHI3L1 in differentiating celiac disease from healthy controls, with AUC values of approximately 0.70. The optimal cut-off values derived from the Youden index provided high sensitivity but moderate specificity, particularly for zonulin, suggesting that these biomarkers may be more useful as supportive screening tools rather than standalone diagnostic markers. Although CHI3L1 showed a diagnostic performance comparable to zonulin, it was not identified as an independent predictor in multivariate analysis, whereas zonulin remained independently associated with disease activity. This difference may reflect the closer relationship of zonulin with intestinal barrier dysfunction, a central mechanism in celiac disease pathogenesis.

The similar AUC values of zonulin and CHI3L1 likely reflect their involvement in interconnected pathophysiological pathways, including epithelial barrier disruption and innate immune activation. However, their moderate discriminative capacity indicates that they cannot replace established serological markers such as tTG-IgA. Instead, these biomarkers may provide complementary information, particularly in early-stage disease, clinically ambiguous cases, or in the assessment of persistent low-grade mucosal inflammation. Since the proposed cut-off values were derived from a single cohort using the Youden index, external validation in larger and independent populations is required before routine clinical application.

### 4.6. Strengths and Limitations

The present study has several limitations. First, its cross-sectional design precluded the assessment of longitudinal changes in biomarker levels within the same individuals over time. Second, although rigorous pre-analytical standardization was applied, including strict sample handling and storage procedures, residual biological variability cannot be entirely excluded. In addition, zonulin levels were measured using commercially available ELISA-based assays, which may lack specificity for pre-haptoglobin 2, the originally described zonulin protein, and may exhibit cross-reactivity with structurally related proteins. This methodological limitation should be considered when interpreting absolute zonulin concentrations.

The relatively low-to-moderate correlation coefficients observed between biomarkers and clinical parameters likely reflect the multifactorial and heterogeneous nature of celiac disease. Unlike acute-phase reactants, biomarkers associated with intestinal permeability and chronic mucosal inflammation may demonstrate slower temporal dynamics and may vary according to the degree of mucosal healing. Nevertheless, the consistent direction of correlations and significant AUC values suggest that zonulin and CHI3L1 may still provide complementary information for detecting persistent or subclinical inflammatory activity.

Future longitudinal studies are needed to clarify temporal relationships of these biomarkers with disease progression and treatment response, potentially enhancing personalized monitoring strategies in CD.

This study has several limitations. First, its cross-sectional design did not allow assessment of longitudinal changes in biomarker levels over time. Second, despite rigorous pre-analytical standardization, residual biological variability cannot be completely excluded. In addition, zonulin was measured using commercially available ELISA assays, which may lack specificity for pre-haptoglobin 2 and may cross-react with structurally related proteins; therefore, absolute zonulin concentrations should be interpreted with caution.

The relatively low-to-moderate correlations observed between biomarkers and clinical parameters may reflect the heterogeneous nature of celiac disease and differences in mucosal healing. Nevertheless, the consistent direction of correlations and significant AUC values suggest that zonulin and CHI3L1 may still provide complementary information regarding persistent or subclinical inflammatory activity.

Despite these limitations, the inclusion of four clinically distinct groups provided a robust framework for evaluating zonulin and CHI3L1 across different stages of disease activity and dietary adherence. To our knowledge, this is also the first pediatric study to evaluate these biomarkers concurrently in relation to both clinical and histopathological findings. Future longitudinal and multicenter studies are needed to clarify their relationship with disease progression, mucosal healing, and treatment response, and to determine their potential role in personalized monitoring strategies in celiac disease.

## 5. Conclusions

Serum zonulin and CHI3L1-3-like protein 1 levels reflect intestinal permeability and inflammatory activity in pediatric celiac disease and are associated with disease activity rather than micronutrient status. These biomarkers may provide complementary information during follow-up, particularly in patients with persistent symptoms or incomplete mucosal recovery despite adherence to a gluten-free diet.

## Figures and Tables

**Table 1:** Comparison of Hematologic, Micronutrient, and Disease-Specific Biomarkers Across the Study Groups.

Parameter	Control (*n* = 42)	Newly Diagnosed CD (*n* = 35)	GFD-Adherent (*n* = 58)	GFD-Nonadherent (*n* = 38)	*p*-Value
**Demographic and Anthropometric Parameters**					
Age (years)	12.0 ± 4.5	9.3 ± 4.1	10.8 ± 4.0	11.6 ± 4.5	0.071 *
Female sex, *n* (%)	27 (62.8)	21 (61.8)	37 (63.8)	26 (68.4)	0.893 †
BMI-SDS	0.19 ± 1.56	−0.67 ± 1.37	−0.89 ± 1.24	−0.68 ± 1.47	0.008 *
**Hematologic Parameters**					
Hemoglobin (g/dL)	13.8 ± 1.3	12.7 ± 1.4	13.0 ± 1.5	12.6 ± 1.9	0.004
Hematocrit (%)	42.0 ± 3.4	38.9 ± 3.5	39.6 ± 4.2	39.3 ± 4.4	<0.001
MCV (fL)	82.9 ± 4.5	79.8 ± 6.1	83.0 ± 5.4	80.6 ± 7.8	0.039
MCHC (g/dL)	32.7 ± 1.2	32.0 ± 2.1	32.6 ± 1.5	31.7 ± 2.1	0.025
Platelet (×10^3^/µL)	313.7 ± 77.4	332.7 ± 86.5	319.3 ± 91.4	333.3 ± 89.2	0.016
WBC (×10^3^/µL)	7.6 ± 1.8	7.9 ± 2.2	7.1 ± 1.9	7.9 ± 2.2	0.131
Neutrophils (×10^3^/µL)	3.9 ± 1.6	3.6 ± 1.7	3.5 ± 1.2	4.2 ± 1.9	0.160
Lymphocytes (×10^3^/µL)	2.9 ± 0.9	3.4 ± 1.3	2.8 ± 1.1	2.9 ± 1.1	0.093
Monocytes (×10^3^/µL)	0.6 (0.2–7.0)	0.6 (0.2–1.2)	0.5 (0.2–7.1)	0.6 (0.3–1.3)	0.068
Eosinophils (×10^3^/µL)	0.2 (0.0–1.0)	0.2 (0.1–5.3)	0.1 (0.0–4.4)	0.2 (0.0–2.8)	0.247
**Micronutrient and Metabolic Parameters**					
Iron (µg/dL)	75.7 ± 48.3	66.8 ± 40.7	78.2 ± 43.5	73.7 ± 39.2	0.634
Ferritin (ng/mL)	39.9 (8.8–185.0)	17.8 (4.8–86.7)	25.0 (5.3–118.0)	20.0 (3.2–147.0)	0.002
Vitamin D (ng/mL)	20.4 ± 7.6	18.8 ± 7.4	19.8 ± 8.9	17.9 ± 9.2	0.593
Calcium (mg/dL)	9.9 ± 0.3	9.7 ± 0.3	9.6 ± 0.4	9.7 ± 0.3	0.012
Phosphorus (mg/dL)	4.6 ± 0.7	4.7 ± 0.5	4.6 ± 0.6	4.6 ± 0.6	0.842
Magnesium (mg/dL)	2.1 ± 0.2	2.1 ± 0.1	2.0 ± 0.3	2.0 ± 0.1	0.005
PTH (pg/mL)	33.9 ± 11.3	33.7 ± 19.1	32.9 ± 10.4	41.3 ± 24.4	0.024
Vitamin B12 (pg/mL)	384 ± 211	397 ± 164	455 ± 168	428 ± 152	0.205
Folate (ng/mL)	9.6 ± 7.1	8.2 ± 4.3	10.1 ± 4.0	7.6 ± 3.8	0.072
**Disease Activity Biomarkers**					
tTG-IgA (U/mL)	1.2 (0.3–12.0)	122.0 (0.8–200.0)	3.0 (0.2–945.0)	40.2 (5.6–200.0)	<0.001
Zonulin (ng/mL)	2.6 (0.5–16.5)	7.7 (0.9–53.4)	5.3 (0.0–52.2)	7.1 (0.3–50.9)	<0.001
CHI3L1 (pg/mL)	306 (14–3219)	1014 (0–6465)	487 (11–5585)	829 (0–4712)	<0.001

Data are presented as mean ± SD, median (range), or number (%). * One-way ANOVA was used for continuous variables. † Chi-square test was used for categorical variables. *p* < 0.05 was considered statistically significant. Abbreviations: BMI-SDS, body mass index standard deviation score; MCV, mean corpuscular volume; MCHC, mean corpuscular hemoglobin concentration.

**Table 2:** Correlation Analysis of Serum Zonulin and Chitinase-1(CHI3L1) with Clinical, Histological, and Laboratory Parameters.

Parameters	Zonulin (r_s_)	*p*-Value	Chitinase-1 (r_s_)	*p*-Value
**Demographic & Anthropometric**				
Age	−0.016	0.833	−0.078	0.310
BMI (Body Mass Index)	0.001	0.986	−0.079	0.299
**Disease Activity Markers**				
Marsh Score (Histopathology)	0.289	<0.001 **	0.243	0.001 **
tTG-IgA (Tissue Transglutaminase)	0.245	0.001 **	0.264	<0.001 **
**Anemia & Iron Profile**				
Hemoglobin	−0.258	0.001 **	−0.160	0.038 *
Hematocrit (Hct)	−0.188	0.014 *	−0.145	0.061
Serum Iron	−0.203	0.007 **	−0.064	0.400
Ferritin	−0.202	0.009 **	−0.078	0.319
MCV	−0.146	0.060	−0.130	0.094
MCHC	−0.182	0.019 *	−0.170	0.029 *
**Vitamins & Minerals**				
Vitamin B12	−0.028	0.718	0.090	0.241
Folate	−0.068	0.395	−0.180	0.023 *
Vitamin D	−0.039	0.617	−0.031	0.691
Calcium	−0.068	0.382	−0.106	0.169
Phosphorus	−0.039	0.610	−0.099	0.201
Magnesium	−0.102	0.189	−0.066	0.401
**Hormonal Markers**				
Parathyroid Hormone (PTH)	0.203	0.010 **	0.190	0.015 *
**Inflammatory Cell Counts**				
WBC (White Blood Cell Count)	0.102	0.185	−0.024	0.758
Lymphocytes	−0.012	0.878	0.052	0.499
Monocytes	0.154	0.046 *	0.022	0.776
PLT (Platelet Count)	0.080	0.299	−0.092	0.236

Spearman correlation analysis was performed. r_s_ indicates Spearman’s correlation coefficient. * *p* < 0.05, ** *p* < 0.01.

## Data Availability

The data presented in this study are available on request from the corresponding author.

## Supplementary Materials

The following supporting information can be downloaded at: https://www.mdpi.com/article/10.3390/children13060730/s1, Table S1: Zonulin and Chitinase-1 (CHI3L1) Levels According to Marsh Classification.

## Abbreviations

The following abbreviations are used in this manuscript:

AUC	Area under the curve
BMI	Body mass index
CD	Celiac disease
CHI3L1	Chitinase 3-like protein 1
CRP	C-reactive protein
ELISA	Enzyme-linked immunosorbent assay
GFD	Gluten-free diet
IBD	Inflammatory bowel disease
IFN-γ	Interferon-gamma
IL-15	Interleukin-15
IL-17A	Interleukin-17A
MCV	Mean corpuscular volume
MCHC	Mean corpuscular hemoglobin concentration
PTH	Parathyroid hormone
ROC	Receiver operating characteristic
SDS	Standard deviation score
tTG-IgA	Tissue transglutaminase immunoglobulin A
WBC	White blood cell count
