# Supplementary material for: Serum Zonulin and Chitinase (CHI3L1) as Biomarkers of Intestinal Permeability and Disease Activity in Pediatric Celiac Disease
Source: Children (Basel). 2026 May 24;13(6):730. doi: 10.3390/children13060730 (PMC13298287; doi:10.3390/children13060730)
Supplement: Supplementary file 1 [file children-13-00730-s001.zip › children-4283861-supplementary.pdf]

Supplementary Table S1. Zonulin and Chitinase -1(CHI3L1) Levels According to Marsh Classification (n = 131).

**Marsh Class**	***n***	**Zonulin Median (IQR) (ng/mL)**	**Chitinase Median (IQR) (ng/mL) ***
Marsh 2	30	5.54 (2.24–19.76)	1080 (217–1851)
Marsh 3a	36	7.29 (4.34–14.46)	642 (213–1851)
Marsh 3b	38	5.60 (2.70–10.31)	802 (321–1444)
Marsh 3c	27	6.25 (3.04–10.77)	733 (424–1943)
p value *		0.937	0.843

* Group comparisons were performed using the Kruskal–Wallis test.
